# Chicago Veterinary College Commencement

**Published:** 1891-04

**Authors:** 


*Chicago Veterinary College Commencement.*—The Eighth Annual Class Day Exercises of the Chicago Veterinary College were held Tuesday, March 24, 1891, at Hooley's Theatre, Chicago. The degree of D. V. S., was conferred by Prof. R. J. Withers, upon the following gentlemen

Allen, J. M. Wisconsin; Annand, J. G. Minnesota; Arpke, H. A, Wisconsin; Archibald, R. A. California; Bagnall, W. P. Pennsylvania; Barnett, J. A. Illinois; Barnett, Otis Illinois; Berner, J. S. Minn.; Bone, J. L. Illinois; Borum, S. D. Illinois; Bristow, G. E. Illinois; Cain, G. E. Massachusetts; Cann, C. Kentucky; Chandler, T. W. Massachusetts; Ditewig, Geo. Illinois; Donaldson, R. H. Minneapolis; Drummond, R. H. Kansas; Depson, O. E. Illinois; Eels, T. C. Illinois; Eisenhower, A. L. Kansas; Fickert, A. F. Jr., Wisconsin; Finley, R. W. Illinois; Fisher, H. S. Illinois; Eitch, D. N. Indianapolis; Fitch, Schuyler, Indianapolis; Folts, D. R. Wisconsin; Fox, D. F. Illinois; French, A. H. Alabama; Gansel, B. E. Illinois; Givinn, E.E. Illinois; Hanna, A. R. Illinois; Harrington, W. B. Illinois; Hawley, H. W. Wisconsin; Heer, R. S. Illinois; Hinckley, E. R. Ohio; Hoover, Lee, Illinois; Johnson, H. J. Wisconsin; Judson, F. E. Illinois; Kelso, J. R. Wisconsin; Kerr, C. E. Illinois; Kinsey, G. W. Michigan; Kinyon, B. F. Wisconsin; Knight, W. A. Kansas; Laws, J. P. Illinois; Lames, R. Iowa; Leech, G. E. Wisconsin; Lennon, J. F. Illinois; Mabie, W. A. Indianapolis; Malone, J.H. Illinois; McNair, E. Illinois; Miller, Fred. Indianapolis; Mohlenhoff, R. H. Illinois; Mohney, C. E. Michigan; Montgomery, C. C. Illinois; Noble, G. E. Iowa; Quitman, E. L. Illinois; Richardson, W. J. Michigan; Rishel, E. H. Michigan; Robinson, W. P. Kansas; Rush, E. G. Ohio; Ryan, J. H. Illinois; Salusbury, H. H. Mississipi; Schaefer, V. Nebraska; Scott, C. E. Illinois; Seago, O. A. Illinois; Seaman, W. A. Illinois; Siegrosser, J. L. Illinois; Shipley, L. N. Iowa; Sherwood, A. E. Iowa; Smith, C. P. Illinois; Spencer, H. T. California; Stull, C. M. Indianapolis; Syler, J. L. Illinois; Watson, J. G. Illinois; Williams, C. Iowa; Wilmington, Jos. Illinois; Yeager, T. D. Illinois.

The Class Officers were :

President, W. A. Seaman; First Vice-President, Geo. Deitwig; Second Vice-President, R. S. Heer; Treasurer, R. A. Archibald; Secretary, Ora E. Dyson.

The Committee of Arrangements consisted of R. H. Donaldson, C. E. Kerr, H. F. Spencer, F. C. Eells, J. F. Lennon, C. E. Scott, A. R. Hanna, J. H. Ryan, J. L. Siegrosser.

